# Supplementary material for: The Promoter Region of the Proto-Oncogene MST1R Contains the Main Features of G-Quadruplexes Formation
Source: Int J Mol Sci. 2022 Oct 26;23(21):12905. doi: 10.3390/ijms232112905 (PMC9653784; doi:10.3390/ijms232112905)
Supplement: Supplementary file 1 [file ijms-23-12905-s001.zip › ijms-1957170-supplementary.pdf]

The promoter region of the proto-oncogene MST1R contains the main features of G-quadruplexes formation

Coralie Robert[1,2, †], Julien Marquevielle[1,2, †] and Gilmar F. Salgado[1,2,*]

1 ARNA laboratory, Inserm U1212, CNRS UMR 5320, University of Bordeaux, 146 Rue Léo Saignat, F-33000 Bordeaux, France.

2 Institut Européen de Chimie et Biologie, UAR 3033 US001, CNRS, University of Bordeaux, 2 Rue Robert Escarpit, F-33600 Pessac, France.

* Correspondence: gilmar.salgado@u-bordeaux.fr

† These authors contributed equally to this work

Supplementary information

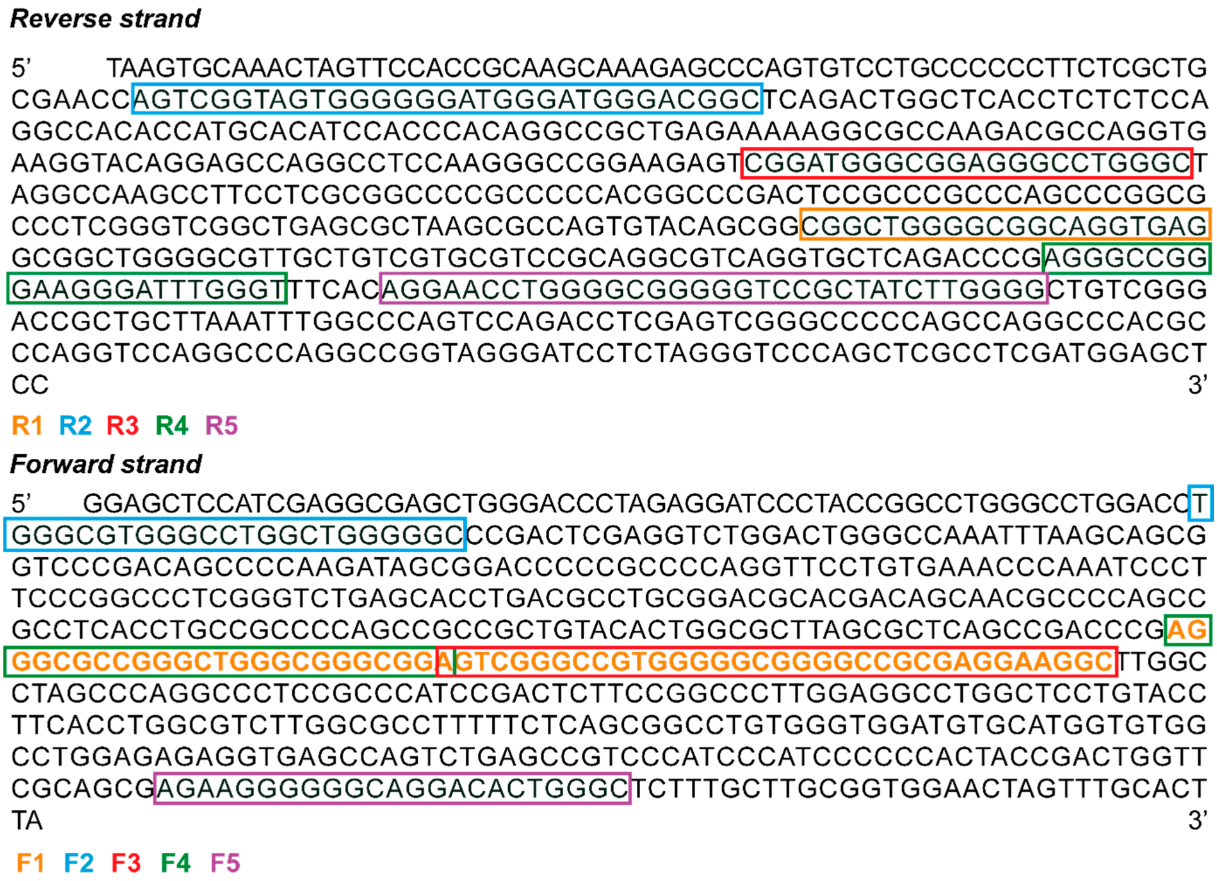

Figure S1. MST1R promoter region with G4 hunter software highest score sequences highlighted in color.

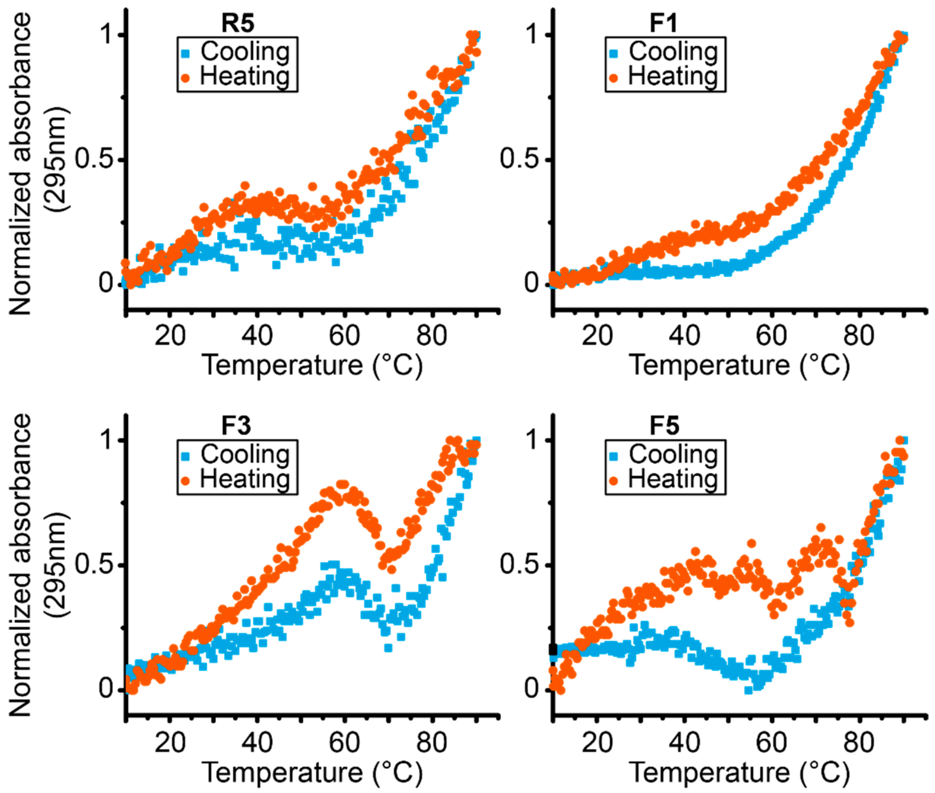

Figure S2. UV-melting spectra of reverse and forward MST1R G-quadruplexes non-forming sequences at 295 nm from 10°C to 90°C. All experiments were performed in buffer 1X (50 mM KCl; 10 mM KPi; pH 6.66).

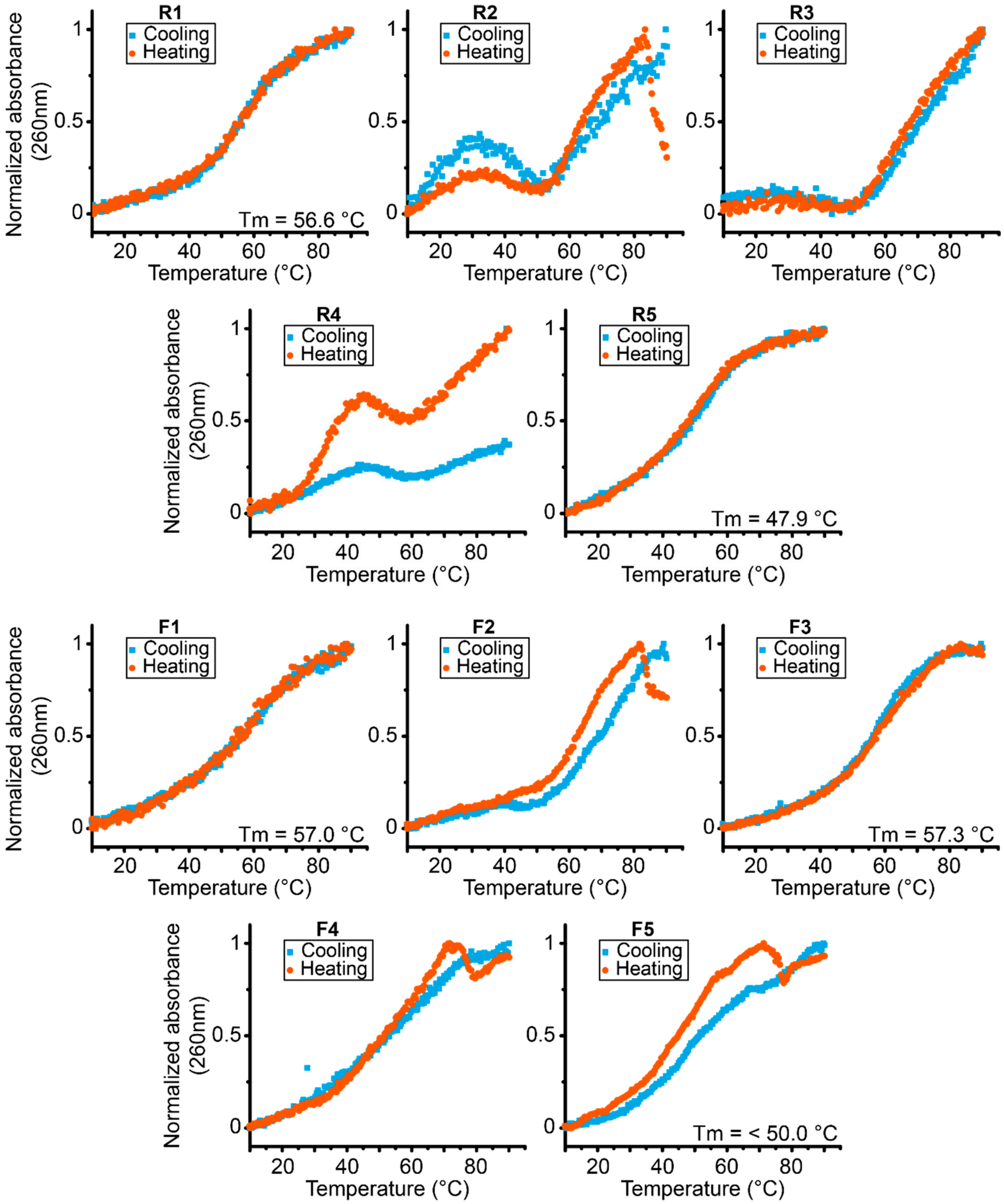

Figure S3. UV-melting spectra of reverse and forward MST1R sequences at 260 nm from 10°C to 90°C. All experiments were performed in buffer 1X (50 mM KCl; 10 mM KPi; pH 6.66).
